# Investigation of the role of sleep and physical activity for chronic disease prevalence and incidence in older Irish adults

**DOI:** 10.1186/s12889-022-14108-6

**Published:** 2022-09-09

**Authors:** Belinda Hernández, Siobhán Scarlett, Frank Moriarty, Roman Romero-Ortuno, Rose Anne Kenny, Richard Reilly

**Affiliations:** 1 The Irish Longitudinal Study On Ageing, Department of Medical Gerontology, School of Medicine, Trinity College Dublin, Dublin, Dublin 2 Ireland; 2 Department of General Practice, HRB Centre for Primary Care Research, Royal College of Surgeons in Ireland, Dublin, Ireland; 3 Mercer’s Institute for Successful Ageing, St. James’s Hospital, Trinity College, The University of Dublin, Dublin, Ireland; 4 School of Engineering, Trinity College, The University of Dublin, Dublin, Ireland; 5 Trinity Centre for Biomedical Engineering, Trinity College, The University of Dublin, Dublin, Ireland

**Keywords:** Multimorbidity, Physical activity, Sleep, Chronic illness

## Abstract

**Background:**

Chronic diseases are the leading cause of death worldwide. Many of these diseases have modifiable risk factors, including physical activity and sleep, and may be preventable. This study investigated independent associations of physical activity and sleep with eight common chronic illnesses.

**Methods:**

Data were from waves 1, 3 and 5 of The Irish Longitudinal Study on Ageing (*n* = 5,680). Inverse probability weighted general estimating equations were used to examine longitudinal lifetime prevalence and cumulative incidence of self-reported conditions.

**Results:**

Sleep problems were significantly associated with increased odds of incident and prevalent arthritis and angina. Additionally sleep problems were associated with higher odds of lifetime prevalence of hypertension and diabetes. Physical activity was negatively associated incident osteoporosis and respiratory diseases and negatively associated with lifetime prevalence of hypertension, high cholesterol and diabetes.

**Conclusions:**

Worse sleep quality and lower physical activity were associated with higher odds of chronic diseases. Interventions to improve sleep and physical activity may improve health outcomes.

**Supplementary Information:**

The online version contains supplementary material available at 10.1186/s12889-022-14108-6.

## Introduction

Chronic non-communicable diseases are the leading cause of death worldwide [1]. In Ireland, approximately one million people live with diabetes, asthma, severe respiratory illness or cardiovascular disease [2]. Many of these chronic illnesses have modifiable risk factors such as overweight and obesity, low physical activity, smoking, alcohol intake and poor diet, which can be prevented with appropriate interventions [3–12]. The onset of such non-communicable diseases in general is related to lower quality of life, mortality and higher burden on healthcare systems [13, 14]. The financial burden of adult obesity alone in Ireland is €1.13 billion per annum [15], while the economic burden of sleep problems resulting from increased healthcare expenses was estimated to be $160 million in Australia in 2016–2017 [16].

Sleep problems become increasingly prevalent in older ages and are commonly reported by those with cardiovascular disease, respiratory illness, obesity, and comorbid medical conditions [17]. Sleep deprivation has been shown to increase blood pressure, inflammation and influence cortisol secretion with implications for physical health [18–21]. Physical activity declines in older ages, but is effective in improving physical outcomes as well as sleep habits [22, 23]. Sleep quality however is facilitating factor in the desire to engage in physical activity, and while physical activity may improve sleep, engagement may also rely on quality of sleep [24, 25]. A collaborative intervention approach to improving sleep and physical activity behaviours may be an effective method of preserving physical health in older adults. To date however, little is known about the independent association of sleep and physical activity with chronic medical conditions.

This study aimed to investigate the independent association between physical activity and sleep quality on eight common medical conditions, in adults aged over 50 in Ireland. This information may be useful in preparing public policy to help limit the burden of chronic illness on our health care system and improve health outcomes for our ageing population.

## Methods

Longitudinal analysis was based on waves 1, 3 and 5 of The Irish Longitudinal Study on Ageing (TILDA) conducted in 2009–2011, 2014–2015 and 2018 respectively. TILDA is a prospective nationally representative study of community dwelling older adults aged 50 and over living in the Republic of Ireland. Regarding the design of the TILDA study; an initial multi-stage probability sample of 640 clusters of residential addresses was obtained from the Irish Geodirectory. Clusters were stratified according to socio-economic status and selected randomly with a probability of selection proportional to the estimated number of persons aged 50 or over in each cluster. The second stage of the sampling procedure involved a random selection of 40 residential addresses from each of the 640 clusters. The design of the TILDA survey has been comprehensively described elsewhere [26, 27].

### Outcome variables

We examined the cumulative incidence and lifetime prevalence of the following eight self-reported physician diagnosed conditions: hypertension, high cholesterol, diabetes, angina, heart attack, respiratory illness (asthma or chronic lung disease), arthritis and osteoporosis. The criteria for hypertension, high cholesterol, diabetes, respiratory illness (asthma/chronic lung disease) and osteoporosis included anyone who reported ever being diagnosed with these conditions or who reported using medications used to treat these conditions based on their WHO Anatomical Therapeutic Classification system codes (see Additional file 1: Appendix 1 and Supplementary Table S1 for a detailed description). Medications were not used to identify heart attack, angina, and arthritis due to lack of pharmacological treatments that would specifically identify individuals with these conditions.

### Independent variables

The independent variables of interest to this study were sleep problems and self-reported physical activity measured using the International Physical Activity Questionnaire which has previously been validated across twelve countries [28]. Respondents answered seven questions regarding the frequency and duration of vigorous, moderate and walking activities in the preceding week. Respondents were then classified as engaging in low, moderate or vigorous activity based on the weekly metabolic equivalents (MET) minutes of moderate to vigorous physical activity as per the IPAQ protocol [29].

To measure sleep problems, participants were asked about their experience of daytime sleepiness on a four-point Likert scale as well as trouble falling asleep and trouble waking up too early, measured on a three-point Likert scale [30]. Items were summated to derive a sleep problem score ranging from 0–7, with higher scores representing greater magnitudes of sleep problems.

### Covariates

Other covariates controlled for were age, sex and education level, BMI, baseline waist hip ratio, self-reported smoking status, delayed memory recall score, chronic pain, the number of comorbidities associated with each medical condition and disabilities. Number of comorbidities were calculated from a total list of 20 self-reported medical conditions. Level of disability was measured using binary variables to indicate difficulties with an instrumental activity of daily living (IADL) (using the telephone, managing money, taking medication, shopping, and preparing meals) and difficulties with any activity of daily living (ADL) (walking across the room, dressing, bathing, eating, getting in or out of bed, and using the toilet).

### Statistical analysis

To estimate disease incidence (i.e. excluding participants who had the relevant outcome at baseline) and population prevalence among survivors at each time point we employed inverse probability weighted generalised estimating equations (IPW-GEE) with an independence working correlation matrix which fully conditions on attrition due to death and includes time of death in the missingness models using the fully conditional IPW estimate proposed in [31]. The IPW estimate also accounts for survey weighting to give valid population inference on disease prevalence and incidence. All analysis was performed using R 4.1.1. Inverse probability models were developed by the authors and IPW-GEE was implemented using the R package GEEpack for more information see Additional file 1: Supplementary Material Appendix 2.

Other alternatives to the GEE marginal models were considered such as linear mixed models with individual random effects and joint models which simultaneously combine a longitudinal and survival model. The work in [32] and in [33] show that linear mixed models implicitly impute post death outcomes when missing data are present due to death and so can be biased. Joint models can fully model both missingness mechanisms and are an equally valid alternative to estimating population level disease prevalence, however as death only occurred in 10.9% of our cohort the marginal IPW-GEE model conditioning on death was selected for parsimony and ease of interpretation.

Variables controlled for in the missingness models were age, sex, education, marital status, delayed recall, immediate recall, animal naming score, smoking history, physical activity intensity, BMI, timed up and go speed, self-reported health, self-reported vision, time of death given survival to current wave as well as the number of: medications, symptoms of depression, disabilities, cardiovascular diseases and chronic diseases.

A directed acyclic graph was used to inform the choice of covariates and to identify a minimal sufficient set of confounders in the IPW-GEE model (see Additional file 1: Appendix 3 Supplementary Figure S1). The directed associations assumed between these variables were based on evidence from the literature and/or expert clinical opinion. For brevity and given that the majority of the covariates included in this analysis are shared risk factors for many non-communicable diseases the same underlying graph structure was assumed for all eight conditions.

Multicollinearity among the model covariates was assessed using the Generalised Variable Inflation Factor (GVIF) which is recommended in the presence of categorical variables. A value of $GVIF 1/2df > 2.24$ was considered as indicative of high multicollinearity, where df is the number of degrees of freedom in a categorical variable. This is equivalent to the widely accepted value of a variable inflation factor value of 5 [34]. High multicollinearity was not found in any of the models investigated.

#### Mediation analysis

To further investigate the potential of sleep quality (measured through the sleep problem score) as a mediator between physical activity level and disease prevalence after controlling for all other covariates mentioned above, a causal mediation analysis was conducted using the mediation package in R4.1.1. The IPW-GEE model previously described was the outcome model. A weighted linear model regressing the sleep problem score on exercise after controlling for all other covariates included in the outcome model was used as the mediation model which assessed the relationship between the sleep problem score and physical activity.

In each case the total effect of physical activity on disease prevalence is decomposed into two measures: the average direct effect (ADE) sometimes referred to as the natural direct effect and the average causal mediation effect (ACME) also known as the natural indirect effect. The ADE measures the expected reduction in the probability of disease that is due to physical activity alone and which does not depend on the sleep problem score after controlling for all other confounding variables. The ACME measures the expected reduction in the probability of disease which is dependent on/mediated by the sleep problem score.

## Results

Table 1 shows a summary of participant characteristics at baseline and Table 2 shows the unadjusted prevalence and incidence of the eight disease outcomes investigated. A total of 3894 participants attended and had valid data across all three waves. Further information on the missingness model and drop out rates can be found in Additional file 1: Appendix 4.

**Table 1:** Characteristics of the TILDA sample at baseline wave 1

Characteristic	Summary
Physical Activity n (weighted %)	
*Low*	1687 (30.02%)
*Moderate*	2003 (34.56%)
*Vigorous*	1990 (35.42%)
Sleep Problems mean (sd)	2.16 (1.64)
Age mean (sd)	62.72 (9.03)
Sex Female n (weighted %)	3082 (49.56%)
Education n (weighted %)	
*Primary or less*	1446 (25.43%)
*Secondary*	2336 (41.32%)
*Third Level*	1898 (33.25%)
Smoking History n (weighted %)	
*Never*	2562 (44.40%)
*Former*	2224 (40.48%)
*Current*	894 (15.12%)
Delayed Recall Score mean (sd)	6.06 (2.29)
Disabilities n (weighted %)	
ADLs n (%)	454 (8.57%)
IADLs n (%)	321 (5.61%)
Chronic Pain n (weighted %)	2080 (36.86%)

**Table 2:** Unadjusted lifetime prevalence and cumulative incidence of the eight disease outcomes investigated

	**Lifetime Prevalence** **Cases/Persons at risk** **(weighted %)**			**Incidence** **Cases/Persons at risk (weighted %)**	
	**Wave 1**	**Wave 3**	**Wave 5**	**4-Year**	**8-Year**
**Hypertension**	2390/5680 (43.82%)	2524/4814 (53.56%)	2255/3950 (61.11%)	545/2835 (19.70%)	716/2411 (32.12%)
**High Cholesterol**	2733/5680 (48.67%)	2977/4814 (62.17%)	2688/3950 (69.13%)	650/2487 (26.15%)	808/2068 (39.35%)
**Diabetes**	423/5680 (8.34%)	471/4814 (10.16%)	455/3950 (12.74%)	130/4473 (2.94%)	195/3690 (5.45%)
**Respiratory Diseases**	757/5680 (13.42%)	803/4814 (16.99%)	743/3950 (20.25%)	186/4197 (4.59%)	250/3206 (8.16%)
**Arthritis**	1578/5680 (28%)	1878/4814 (39.9%)	1750/3950 (47.63%)	557/3493 (16.25%)	711/2911 (26.16%)
**Osteoporosis**	370/5680 (10.27%)	1002/4814 (20.96%)	1002/3950 (26.36%)	434/4246 (10.22%)	547/3493 (16.07%)
**Angina**	291/5680 (5.29%)	283/4814 (6.15%)	240/3950 (7.21%)	65/4596 (1.49%)	95/3805 (2.91%)
**Heart Attack**	254/5680 (4.79%)	264/4814 (5.72%)	215/3950 (6.5%)	68/4618 (1.55%)	80/3815 (2.59%)

### Cumulative incidence

The odds ratios and 95% confidence intervals for the models of cumulative incidence of disease can be seen in Table 3. Covariates were non-time varying and taken at their baseline values. Here it can be seen that baseline vigorous activity was associated with 18% lower odds of incident osteoporosis (*p*-value 0.041) and marginally associated with 24% lower odds of incident respiratory diseases (*p*-value 0.047). Regarding sleep problems, a one unit increase in baseline sleep problem score was marginally associated with 5% increased odds of incident arthritis (*p*-value 0.046) and 11% increased odds of angina (*p*-value 0.047). In all cases models controlled for age, sex, education, BMI, waist hip ratio (WHR), smoking status, physical activity, cognition, disabilities, chronic pain and comorbidities.

**Table 3:** Odd Ratios (OR) and 95% confidence intervals (95% CI) for the models of cumulative disease incidence with respect to physical activity and sleep problems

	***Physical Activity*** ***OR (95% CI)***		***Sleep Problems*** ***OR (95% CI)***
	*Moderate*	*Vigorous*	
**Hypertension**	0.88 (0.734–1.06)	0.84 (0.70–1.00)	1.02 (0.98–1.07)
**High Cholesterol**	1.01 (0.85–1.21)	0.97 (0.81–1.15)	1.04 (0.99–1.08)
**Diabetes**	1.15 (0.85–1.56)	0.94 (0.69–1.28)	0.99 (0.91–1.06)
**Respiratory Diseases**	1.03 (0.8–1.33)	0.76 (0.58–0.99)^*^	1.02 (0.95–1.09)
**Arthritis**	1.13 (0.95–1.36)	1.13 (0.95–1.35)	1.05 (1.00–1.09)^*^
**Osteoporosis**	0.98 (0.81–1.18)	0.82 (0.67- 0.99)^*^	1.01 (0.96–1.06)
**Angina**	0.71 (0.47–1.06)	0.67 (0.43–1.06)	1.11 (1.00–1.24)^*^
**Heart Attack**	1.22 (0.79–1.87)	0.90 (0.55–1.48)	1.04 (0.94–1.16)

^*^0.01 < *p-value* ≤ *0.05*

### Lifetime prevalence

#### Sleep problems

**Fig. 1:**
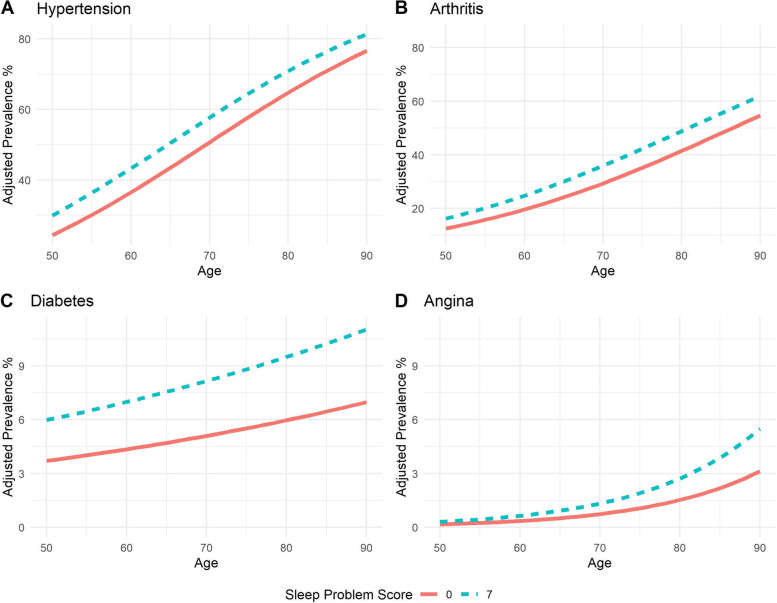
2018 Wave 5 Adjusted prevalence of medical conditions significantly associated sleep disturbance score for the average Irish adult aged 50 + . Solid line indicates the marginal mean estimate for a sleep disturbance score of 0, dashed lines represent marginal mean estimate for a sleep disturbance score of 7. Note: the average Irish adult is a non-smoker with secondary level education, no disabilities, BMI 28.7, waist-hip ratio 0.9, no chronic pain, delayed recall score of 6.08 and engages in moderate physical activity

Lifetime prevalence of the eight conditions was also investigated and it was found that the sleep problem score was significantly associated with lifetime prevalence of hypertension (*p*-value 0.02), arthritis (*p*-value 0.02), diabetes (*p*-value 0.01), and angina (*p*-value 0.01) and marginally associated with respiratory illness (*p*-value 0.048) in fully adjusted models. Figure 1 shows the wave 5 adjusted population prevalence with respect to age for a sleep problem score of 0 versus 7. Additional file 1: Supplementary Tables S2-S9 Appendix 5 show the odds ratios, confidence intervals and *p*-values of the analyses for each of the eight medical conditions.

For hypertension each additional unit of the sleep problem score was associated with increased odds of 1.04 or equivalently increased prevalence of 6.3% for those with a sleep problem score of 0 compared to 7 (prevalence for a score of seven 56.9%; prevalence for a score of zero 50.6%; see Fig. 1A and Additional file 1: Supplementary Table S2). For arthritis, a one unit increase in the sleep problem score was associated with an increased prevalence of 4.9% in males and 6.2% in females for those with a sleep problem score of 7 compared to 0 (Additional file 1: Supplementary Table S3, Fig. 1B).

With respect to diabetes each additional unit increase in the sleep problem score was associated with increased odds of 7% resulting in an increased adjusted population prevalence of 3.09% from those with a score of 0 compared to 7 (Fig. 1C, Additional file 1: Supplementary Table S4). For angina the adjusted prevalence for the average older Irish adult was 2.13% higher in males (5.02%,2.89% for problem scores of 7 and 0 respectively) and 0.81% higher in females (1.85%,1.04% for sleep problem scores of 7 and 0 respectively) see Fig. 1D and Additional file 1: Supplementary Table S5.

There was marginal evidence for an association between respiratory illness and sleep problem score (*p*-value 0.048, see Additional file 1: Supplementary Table S6). For respiratory illness the adjusted prevalence in those with a sleep problem score of 7 compared to 0 was 3.69% higher in females (prevalence 16.26%, 12.57% respectively) and 2.55% higher in males (prevalence 10.74% and 8.19% respectively).

#### Physical activity

**Fig. 2:**
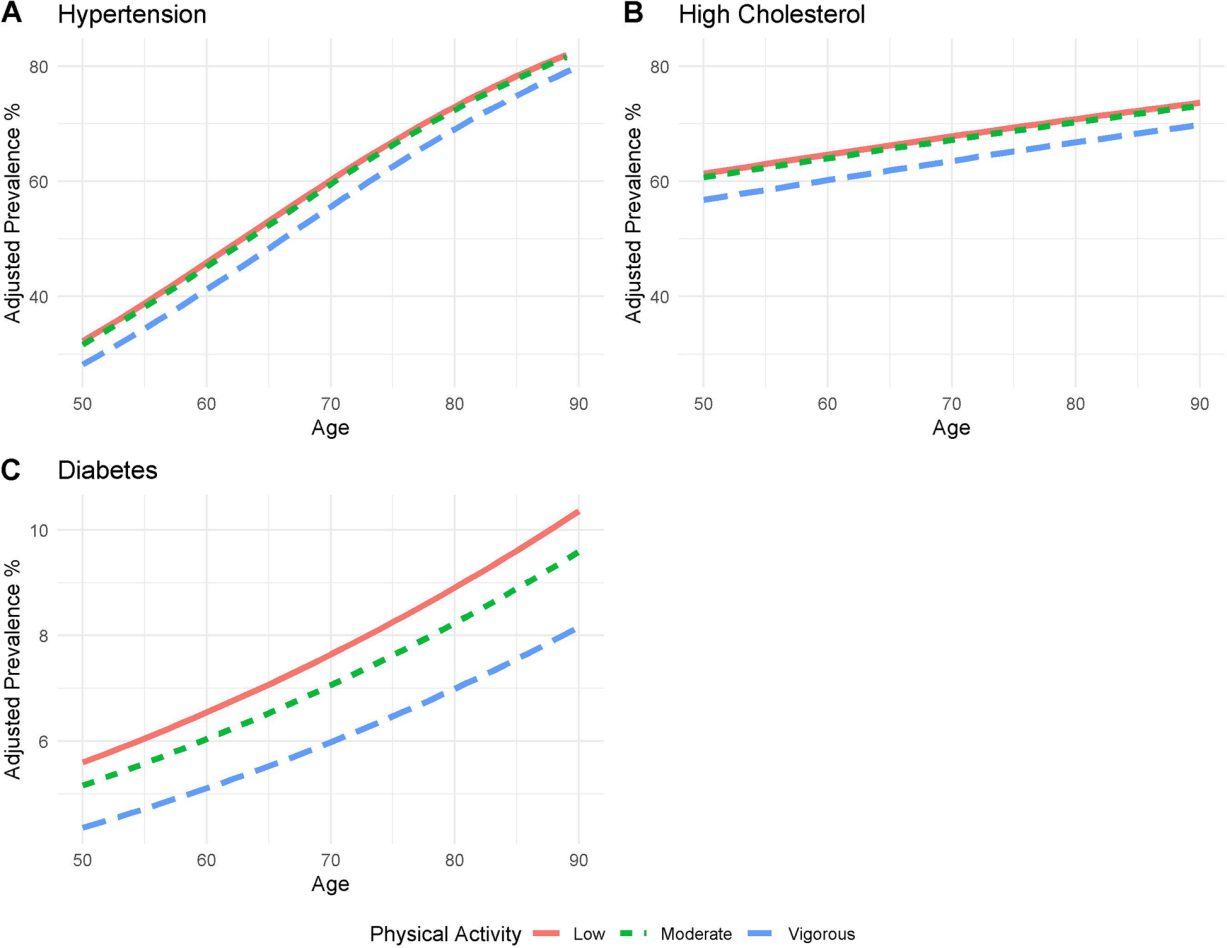
2018 Wave 5 Adjusted prevalence of medical conditions significantly associated with physical activity for the average Irish adult aged 50 + . Solid line indicates the marginal mean estimate for inactive/low physical activity; short dashed lines represent marginal mean estimate for moderate physical activity; long dashed lines represent vigorous physical activity. Note: the average Irish adult is a non-smoker with secondary level education, no disabilities, BMI 28.7, waist-hip ratio 0.9, no chronic pain, delayed recall score of 6.08 and engages in moderate physical activity

Physical activity was significantly negatively associated with hypertension (*p*-value 0.006), high cholesterol (*p*-value 0.003) and diabetes (*p*-value 0.035) in fully adjusted models and was also marginally negatively associated with osteoporosis (*p*-value 0.058). Figure 2 shows the wave 5 adjusted disease prevalence of these conditions with respect to age for low, moderate and vigorous physical activity. Odds of hypertension were associated with a 17.3% decrease for those who engaged in vigorous versus low physical activity (*p*-value 0.004) or equivalently a decrease in adjusted prevalence from 59.2% (low) to 55.0% (vigorous) for an average Irish adult aged over 50 (Additional file 1: Supplementary Table S2, Fig. 2A). With respect to high cholesterol, odds were 17.2% lower for those who engaged in vigorous versus low physical activity (*p*-value 0.002) or an average reduction in prevalence of 4.2% from 67.7% (low) to 63.5% (vigorous) (Additional file 1: Supplementary Table S7, Fig. 2B). Engaging in vigorous versus low physical activity was associated with reduced odds of 25% of diabetes (*p*-value 0.015) reducing the average prevalence from 7.76% (low) to 6.08% (vigorous) (Additional file 1: Supplementary Table S4, Fig. 2C).

There was marginal evidence for the association between osteoporosis and physical activity (*p*-value 0.058, Additional file 1: Supplementary Table S8). Here vigorous physical activity was associated with odds of 0.825 compared with those who engaged in low physical activity or an average prevalence of 32.9% for those who engaged in low physical activity versus 28.9% for those who engage in vigorous physical activity.

There was no evidence for an interaction effect between physical activity and the sleep problem score for any of the conditions investigated.

### Mediation analysis

Additional file 1: Supplementary Table S10 shows the results of the causal mediation analysis for high cholesterol. Here it can be seen that there are significant total, direct and indirect effects for low versus vigorous and moderate versus vigorous physical activity. In particular, the direct effect of vigorous physical activity was on average 3.8% lower than those who engaged in low physical activity. A further 0.1% reduction in high cholesterol was attributable to the sleep problem score. The total effect of moderate versus vigorous physical activity was a reduction of 2.9% in high cholesterol prevalence, 3.3% of which was mediated by the sleep problem score. Low versus moderate physical activity was not significantly associated with a reduction in disease prevalence.

The ADE and ACME for hypertension was -3.8% and -0.001 respectively for low versus vigorous physical activity (see Additional file 1: Supplementary Table S11). For moderate versus vigorous physical activity significant direct and indirect effects were also found of -3.2% and -0.1% respectively. Again, low versus moderate physical activity did not result in any significant effect. For diabetes only low versus vigorous physical activity had significant direct, indirect and total effects of (-2.1% *p*-value 0.012; -0.1% *p*-value 0.012 and -2.2% *p*-value 0.012 respectively, see Additional file 1: Supplementary Table S12). Moderate compared to vigorous and low versus moderate physical activity did not have any significant effect on diabetes prevalence. Similar results were observed for osteoporosis (see Additional file 1: Supplementary Table S13) whereby significant direct, indirect and total effect were found for low versus vigorous physical activity only. The total effect of vigorous as opposed to low physical activity on osteoporosis prevalence was also -2.2%, 4.3% of which was mediated by the sleep problem score. In all cases the mediation and outcome models suggest that higher levels of physical activity, decrease the sleep problem score which in turn reduces disease prevalence. There was no evidence of a moderation effect between physical activity and the sleep problem score.

### Other risk factors

Regarding the lifetime prevalence of the conditions studied; age was significantly positively associated with all but respiratory illness. Smoking history was significantly positively related to all conditions except hypertension, high cholesterol and arthritis. For diabetes, angina, heart attacks, respiratory illness and arthritis having a history of smoking as opposed to never having smoked in general was associated with higher prevalence of disease. Osteoporosis was the only condition associated with lower prevalence for those with a history of smoking. In particular, it was found that current smokers had lower prevalence of osteoporosis than those who never smoked (OR 0.83, *p*-value 0.03). With regards to body composition, BMI was found to be positively associated with diabetes, hypertension, arthritis, angina and heart attacks and negatively associated with osteoporosis whereas waist hip ratio was positively associated with diabetes, hypertension, high cholesterol and respiratory illness. Chronic pain was also associated with higher prevalence of osteoporosis, respiratory diseases and arthritis.

## Discussion

This study revealed two major outcomes 1) poor sleep quality measured through the sleep problem score is inversely associated with cumulative incidence of two out of eight and inversely associated with lifetime prevalence of four out of eight major chronic and cardiovascular medical conditions 2) vigorous physical activity is associated with lower incidence of two out of eight and lower prevalence of three out of eight medical conditions.

In particular, the sleep problem score was significantly associated with both cumulative incidence and lifetime prevalence of angina and arthritis. Additionally sleep problems were also associated with increased odds of diabetes, and hypertension prevalence. With respect to physical activity, engaging in three hours vigorous activity over at least three days per week or 6 h of a combination of walking, moderate and vigorous activity per week was associated with reduced odds of lifetime prevalence of diabetes by 25%, hypertension by 17.3% and high cholesterol by 27.2% and reduced odds of incident osteoporosis by 18.4%. These effects remained significant regardless of age, sex, education, BMI, waist hip ratio (WHR), smoking status, cognition, disabilities, presence of chronic pain and comorbidities. These findings are in line with the literature. In particular, He et al. found that lower physical activity and low sleep duration was associated with prevalence of multimorbidity [35].

Mediation analysis suggested that vigorous as opposed to low physical activity was associated with lower probability of hypertension, high cholesterol, diabetes and osteoporosis prevalence. There was no evidence to suggest that increased physical activity from low to moderate had any effect on disease prevalence. These results also provided evidence to support the hypothesis that higher levels of physical activity in general, improve sleep quality which in turn decreases disease prevalence for all four conditions associated with physical activity.

With respect to the role of sleep quality as a mediator between physical activity and disease prevalence, in all cases the sleep problem score accounted for between 2–4% of the overall effect of physical activity on disease prevalence. There was no evidence that higher levels of physical activity resulted in greater improvements in sleep quality and therefore no evidence of a moderation effect. This suggests that although our results provide evidence to suggest sleep quality plays a minor mediating role between physical activity and disease prevalence; physical activity and sleep quality measured through the sleep problem score are mostly independent mechanisms which contribute to reduced disease prevalence.

Sleep problems have previously been linked to chronic disease in older adults [17, 36, 37]. Foley et al. found that 40% of those with a major comorbidity had fair or poor sleep quality, and sleep disturbances were independently associated with arthritis, lung disease, heart disease and diabetes [17] while Newman et al. showed that older adults with angina were more likely to report trouble falling asleep [37]. Maggi et al. also reported associations between insomnia and arthritis, chronic obstructive pulmonary disease and myocardial infarction [38]. Studies to date using large, population samples however are primarily cross-sectional, and direction of these associations have not been established.

Sleep problems may be improved through intervention strategies with alternative options to use sleep medication [39] showing positive impacts. Sleep hygiene is one approach, promoting healthy behaviours that improve sleep such as regular sleep schedules, avoidance of stimulants and napping, and light exercise [40]. Randomised control trials of cognitive behavioural therapy, simplified sleep restriction, and Tai Chi have also shown improvements in sleep quality [41–45].

Regarding physical activity, vigorous physical activity at baseline was associated with reduced odds of incident respiratory illness and ostroporosis and was also marginally associated with lifetime prevalence of both of these conditions which is in line with the literature [46–48]. For many of the other conditions the number of new incidences were low and so the lack of significance may be due to the analysis being underpowered rather than a lack of association. The direction of the odds in all cases remained as hypothesised.

The sex differences found in this study are in line with previous studies which also show females had significantly higher odds of having osteoporosis, arthritis and respiratory illness and men significantly higher odds of having angina and heart attacks [49]. Crimmins et al. similarly show that arthritis is more prevalent in women at all ages, while men over 50 have a higher prevalence of heart disease [50].

BMI was negatively associated with osteoporosis as shown elsewhere [51]. Contrary to international literature however, we found that having a smoking history was associated with lower odds of osteoporosis [52]. We used physician’s diagnosis of osteoporosis which relies on self-reported data and may not capture the true extent of osteoporosis in this cohort. Bone density measurements are taken in the TILDA health assessment, and high rates of underdiagnosis of osteoporosis were reported [53]. At baseline, 66% of older women, and 100% of older men with objective evidence of osteoporosis did not have a corresponding physician’s diagnosis [53]. Future waves of TILDA with follow-up objective measurements will help to clarify this association.

### Limitations

Some limitations were present. TILDA is a relatively young, healthy sample and incidence of new disease cases may have been too low, to provide statistical power needed for establishing evidence for causal associations in some cases.

Chronic disease prevalence, sleep quality and physical activity were determined by self-reported data. As such, prevalence and incidence of diseases may be underestimated in cases where a participant has not yet engaged with the healthcare system to get a diagnosis, or a participant may not report a chronic disease as the condition has been managed [49]. Regarding the use of self-reported physical activity [54], found that the IPAQ measure typically overestimated physical activity when compared with objective measures. Therefore, it is possible that the effect of physical activity on chronic illnesses is underestimated in this analysis. TILDA collected actigraphy data on a wave 3 sub-sample but this is cross-sectional only at present [27]. Repeated measurements in future waves will provide opportunity to further assess these patterns using longitudinal objectively measured sleep and physical activity.

Regarding attrition, participants who had worse mental and physical health were significantly more likely to drop out of the study. For this reason, an algorithm was developed to weight and correct disease incidence and prevalence estimates for these biases as much as possible. However, it is still possible that our model estimates remain biased due to misspecification of the missingness model or due to the missingness mechanism being missing not at random or put more simply that the probability of attrition may be due to reasons which are unknown and not captured as part of the TILDA study.

The mediation analysis performed requires some strong assumptions two of which are that there is no reverse causality and that all possible confounders have been accounted for. Although, this study used longitudinal data over an 8-year period and our findings are in line with the literature, these results alone cannot definitively conclude a causal association between increased physical activity and improved sleep quality leading to reduced disease prevalence. Reverse causality i.e. the onset of the disease in question causing lower engagement in physical activity and higher sleep problems cannot be categorically ruled out. For example [55] showed that sleep mediated between 11.2% and 43.7% of the association between diabetes, arthritis, asthma, angina and physical activity after disease onset. To account for this as much as possible; poorer general health (comorbidities), poorer physical functioning (ADLs and IADs), lower cognitive ability and presence of chronic pain were controlled for in all cases as these are the likely mechanisms through which onset of many of the chronic and cardiovascular diseases studied could negatively affect physical activity and sleep quality after onset and hence control for reverse causality. To provide further evidence regarding the direction of the association, disease incidence was also investigated.

This is one of the largest studies of trajectories of chronic disease, sleep quality and physical activity in a community dwelling cohort of older adults to date. TILDA collects extensive data at each interview which allowed us to identify these associations while adjusting for a comprehensive list of potential confounding factors. Repeat data was available at four and eight-year follow up, facilitating sophisticated analyses of the chronic disease risk.

These findings show that both sleep problems and physical activity were independently associated with common chronic and cardiovascular conditions. Frequent sleep and activity screening of older adults may highlight those with underlying health conditions or who are at risk of developing chronic or cardiovascular illnesses. Furthermore, intervention strategies can be effective in improving sleep quality and physical activity [42, 43, 45, 56, 57]. This may offer one pathway to target modifiable risk factors for these conditions, limiting the burden of chronic illness on the older population. Policy makers should be aware of a potentially small but mutually beneficial collaboration between intervention strategies targeting improved sleep and physical activity habits.

## Conclusion

Worse sleep quality and lower physical activity were associated with higher odds of chronic disease incidence and prevalence in older adults. Intervention strategies to improve sleep and physical activity may improve health outcomes. Further work is needed to establish whether longitudinal patterns of improved sleep and physical activity habits are effective in preventing the onset of chronic disease.

## Supplementary Information

**Additional file 1: Appendix 1.** Definition of disease outcomes and medications. Supplementary Table S1. Drug classes and corresponding ATC codes used to identify conditions. **Appendix 2.** Supplementary Methods. **Appendix 3.** Supplementary Figure S1. A directed acyclic graph showing the hypothesised relationship between variables. Oval variable are unobserved constructs; rectangles represent observed and measured variables. Physical activity and sleep disturbance score are identified as exposures and disease the outcome variable in each case. **Appendix 4.** Missingness Model. **Appendix 5.** Supplementary Table S2. Results of survey weighted IPW-GEE to predict prevalence of hypertension. Supplementary Table S3. Results of survey weighted IPW-GEE to predict prevalence of arthritis. Supplementary Table S4. Results of survey weighted IPW-GEE to predict prevalence of diabetes. Supplementary Table S5. Results of survey weighted IPW-GEE to predict prevalence of angina. Supplementary Table S6. Results of survey weighted IPW-GEE to predict prevalence of respiratory disease. Supplementary Table S7. Results of survey weighted IPW-GEE to predict prevalence of high cholesterol. Supplementary Table S8. Results of survey weighted IPW-GEE to predict prevalence of osteoporosis. Supplementary Table S9: Results of survey weighted IPW-GEE to predict prevalence of heart attack. **Appendix 6.** Causal Mediation Analysis Results. Supplementary Table S10. High Cholesterol Causal Mediation Results. Supplementary Table S11. Hypertension Causal Mediation Results. Supplementary Table S12. Diabetes Causal Mediation Results. Supplementary Table S13. Osteoporosis Causal Mediation Results. 

## Data Availability

The dataset(s) supporting the conclusions of this article is(are) available in the Irish Social Science Data Archive (ISSDA). For information and to apply to access data from TILDA, please visit www.ucd.ie/issda/data/tilda
