# Supplementary material for: Investigation of the role of sleep and physical activity for chronic disease prevalence and incidence in older Irish adults
Source: BMC Public Health. 2022 Sep 9;22:1711. doi: 10.1186/s12889-022-14108-6 (PMC9463855; doi:10.1186/s12889-022-14108-6)
Supplement: Supplementary file 1 — Additional file 1: Appendix 1. Definition of disease outcomes and medications. Supplementary Table S1. Drug classes and corresponding ATC codes used to identify conditions. Appendix 2. Supplementary Methods. Appendix 3. Supplementary Figure S1. A directed acyclic graph showing the hypothesised relationship between variables. Oval variable are unobserved constructs; rectangles represent observed and measured variables. Physical activity and sleep disturbance score are identified as exposures and disease the outcome variable in each case. Appendix 4. Missingness Model. Appendix 5. Supplementary Table S2. Results of survey weighted IPW-GEE to predict prevalence of hypertension. Supplementary Table S3. Results of survey weighted IPW-GEE to predict prevalence of arthritis. Supplementary Table S4. Results of survey weighted IPW-GEE to predict prevalence of diabetes. Supplementary Table S5. Results of survey weighted IPW-GEE to predict prevalence of angina. Supplementary Table S6. Results of survey weighted IPW-GEE to predict prevalence of respiratory disease. Supplementary Table S7. Results of survey weighted IPW-GEE to predict prevalence of high cholesterol. Supplementary Table S8. Results of survey weighted IPW-GEE to predict prevalence of osteoporosis. Supplementary Table S9: Results of survey weighted IPW-GEE to predict prevalence of heart attack. Appendix 6. Causal Mediation Analysis Results. Supplementary Table S10. High Cholesterol Causal Mediation Results. Supplementary Table S11. Hypertension Causal Mediation Results. Supplementary Table S12. Diabetes Causal Mediation Results. Supplementary Table S13. Osteoporosis Causal Mediation Results. [file 12889_2022_14108_MOESM1_ESM.docx]

**Appendix 1 Definition of disease outcomes and medications**

All medical conditions studied included self-reported doctor diagnosed life-time prevalence the respective medical condition using answers to a question for the following format: “has a doctor ever told you that you have X?”. Self-reported lifetime prevalence of a given disease at a given wave was calculated using responses from all previous waves and corrected for disputes of disease diagnosis between waves. Self-reported medications which were indicative of hypertension, diabetes, high cholesterol osteoporosis and respiratory illness (defined as asthma or chronic lung disease) were also available. Therefore, for these conditions, respondents were identified as having the condition if they either self-reported being diagnosed by a doctor or reported taking medications which are used to treat the identified condition. Medication usage was not used to identify angina, heart attacks or arthritis as it was not possible to identify medications which were specific only to these conditions.

With respect to medications TILDA participants are asked to report prescribed medications, as well as non-prescription medicines and supplements, which they take on a regular basis. Relevant medications were identified based on their WHO Anatomical Therapeutic Classification system codes (Supplementary Table A1). Omega-3 supplements were excluded from the definition of high cholesterol treatments. Asthma or lung disease medications were defined based on maintenance therapies recommended in the Global Initiative for Chronic Obstructive Lung Disease (GOLD) and Global Initiative for Asthma (GINA) guidelines. This excluded those using short-acting beta agonists alone, which may indicate mild disease or symptom management in respiratory infection. High blood pressure medications were defined based on recommendations up to fourth line therapy in the NICE guidelines. Medications were not used to identify heart attack, angina, and arthritis due to lack of pharmacological treatments that would specifically identify individuals with these conditions.

*Supplementary Table S1 Drug classes and corresponding ATC codes used to identify conditions.*

| **Conditions and drug classes** | **Anatomical Therapeutic Classification codes** |
| --- | --- |
| **High blood pressure**  Thiazide diuretics  Alpha-adrenoreceptor antagonists  Selective beta blocking agents  Caclium channel blockers  Angiotensin-converting enzyme (ACE) inhibitors  Angiotensin II receptor blockers (ARBs) | C03A  C02CA C02LE  C07AB C07AG  C08C C08D C08E C08G  C09A C09B  C09C C09D |
| **Diabetes**  Insulins and analogues  Blood glucose lowering drugs (excl. insulins)  Other diabetes drugs | A10A  A10B  A10X |
| **High cholesterol**  Lipid-modifying agents (excl. omega-3-triglycerides) | C10 (excl. C10AX06) |
| **Osteoporosis**  Bisphosphonates  Strontium ranelate  Denosumab  Teriparatide  Parathyroid hormone  Calcitonin preparations  Raloxifene | M05BA M05BB  M05BX03 M05BX53  M05BX04  H05AA02  H05AA01 H05AA03  H05BA  G03XC01 |
| **Asthma or lung disease**  Inhaled corticosteroids  Long-acting beta-agonists  Anti-muscarinics  Antiallergic agents  Xanthines  Leukotriene receptor agonists  Other respiratory agents | R03BA R03AK06 R03AK07 R03AK08 R03AK09 R03AK10 R03AK11 R03AK12 R03AK13  R03AC04 R03AC05 R03AC06 R03AC07 R03AC08 R03AC09 R03AC10 R03AC11 R03AC12 R03AC13 R03AC14 R03AC15 R03AC16 R03AC17 R03AC18 R03AC19 R03AK05 R03AK06 R03AK07 R03AK08 R03AK09 R03AK10 R03AK11 R03AK12 R03AL01 R03AL03 R03AL04 R03AL05 R03AL06 R03AL07 R03AL08 R03AL09  R03BB R03AL  R03BC R03AK04 R03AK05  R03DA  R03DC  R03CC03 R03CB03 R03DX |

**Appendix 2:**

**Supplementary Methods**

Generalized estimating equations [1] are an efficient method for calculating the population level marginal mean of an outcome of interest when the data are longitudinal or clustered. Longitudinal studies of ageing populations often face attrition due to dropout as well as death. Statistical analysis based on complete-cases or fully observed data, may underestimate the true prevalence of a disease as such models are based on a likely healthier than average cohort of survivors who were healthy enough to attend and complete all waves of the study under analysis [2]. Missingness due to death is a distinct mechanism to missingness due to drop out as the values for data post-death do not exist, hence post death missing values must not be imputed or given weight in the final analysis [3, 4]. If missingness due to death is ignored and modelled in the same way as missingness due to drop out, then estimates based on such data will yield an “immortal cohort” inference by either explicitly or implicitly assuming that trajectories continue beyond death and estimate the mean disease prevalence for the entire cohort who are alive and dead [3, 5]. In order to account for missing data due to both missingness mechanisms, we employed inverse probability weighting (IPW) where logistic regression models are used to calculate the probability of each participant being observed at a given wave. Observations are then weighted according to their inverse probability of being observed so that those who had low probability of being observed receive a higher weight. Time of death during this study was known. It was assumed likely that those close to death may be more likely to drop out and so our missingness model fully conditioned on death using the fully conditional IPW estimate ($IPW\_f$) proposed in [6]. In all cases analyses were also survey weighted to give valid population inference on disease prevalence. An IPW-GEE using an independence working correlation matrix was employed as it has been shown this yields unbiased estimates when there is missing data due to death [4, 7]. However, our results were not sensitive to the selection of correlation matrix and conclusions remained unchanged even when a completely unstructured correlation matrix was used.

**References**

1. Liang, K.-Y. and S.L. Zeger, *Longitudinal data analysis using generalized linear models.* Biometrika, 1986. **73**(1): p. 13-22.

2. Little, R.J. and D.B. Rubin, *Statistical analysis with missing data*. Vol. 793. 2019: John Wiley & Sons.

3. Dufouil, C., C. Brayne, and D. Clayton, *Analysis of longitudinal studies with death and drop‐out: a case study.* Statistics in medicine, 2004. **23**(14): p. 2215-2226.

4. Kurland, B.F. and P.J. Heagerty, *Directly parameterized regression conditioning on being alive: analysis of longitudinal data truncated by deaths.* Biostatistics, 2005. **6**(2): p. 241-258.

5. Wen, L. and S.R. Seaman, *Semi‐parametric methods of handling missing data in mortal cohorts under non‐ignorable missingness.* Biometrics, 2018. **74**(4): p. 1427-1437.

6. Wen, L., G.M. Terrera, and S.R. Seaman, *Methods for handling longitudinal outcome processes truncated by dropout and death.* Biostatistics, 2018. **19**(4): p. 407-425.

7. Raitanen, J., et al., *Longitudinal change in physical functioning and dropout due to death among the oldest old: a comparison of three methods of analysis.* European journal of ageing, 2019: p. 1-10.

**Appendix 3**

**
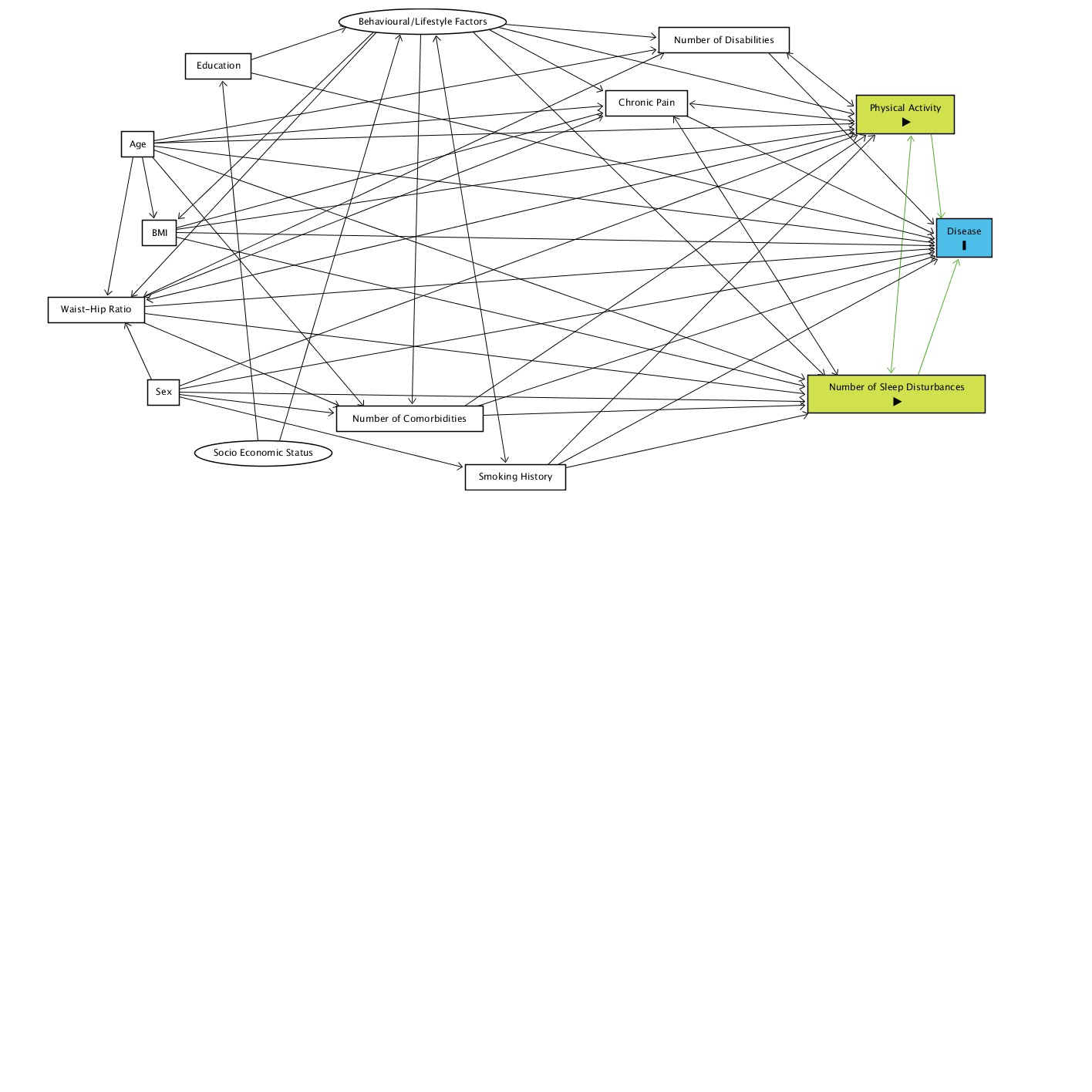
**

**Supplementary** **Figure S1: A directed acyclic graph showing the hypothesised relationship between variables. Oval variable are unobserved constructs; rectangles represent observed and measured variables. Physical activity and sleep disturbance score are identified as exposures and disease the outcome variable in each case**

**Appendix 4**

*Missingness Model*

A total of 5680 TILDA participants who completed the wave 1 computer assisted personal interview (CAPI) and health assessment at baseline 2010 were included in this study. Of 69% (3894) had complete data for all covariates and attended subsequent waves 3 and 5; 14% (807 participants) dropped out after wave 1 and didn’t return; 288 of these were due to death. A further 16% (923) dropped out after wave 3, 333 of whom died. A small number of observations (59) dropped out at wave 3 and later returned in wave 5. Thus, the pattern of missingness was predominantly monotone and IPW estimates were calculated under this assumption. Treating the 59 observations who later returned in wave 5 as non-monotone or excluding them from analysis did not change the results of this study. In general, it was found that participants who had lower delayed memory recall scores, worse animal naming scores, were current smokers, single, taking higher number of medications, had worse self-reported health, higher number of depressive symptoms and higher number of disabilities at baseline were significantly more likely to drop out of the study. Hence showing the need to design appropriate weighting models.

**Appendix 5**

*Supplementary Table S2: Results of survey weighted IPW-GEE to predict prevalence of hypertension*

|  | **Estimate** | **Standard Error** | **p-value** | **95% CI Lower Limit** | **95% CI**  **Upper Limit** |
| --- | --- | --- | --- | --- | --- |
| (Intercept) | **0.000111** | **0.578** | **< 0.001** | **3.57E-05** | **0.000344** |
| Age | **1.06** | **0.00426** | **< 0.001** | **1.05** | **1.07** |
| BMI | **1.09** | **0.00783** | **< 0.001** | **1.07** | **1.11** |
| Waist Hip Ratio | **15.9** | **0.528** | **< 0.001** | **5.66** | **44.9** |
| **Sex:** |  |  |  |  |  |
| Female | 0.926 | 0.0845 | 0.363 | 0.785 | 1.09 |
| **Education:** |  |  | 0.08 |  |  |
| Primary or less  (Baseline) |  |  |  |  |  |
| Secondary | 0.87 | 0.0861 | 0.105 | 0.735 | 1.03 |
| Third Level | **0.816** | **0.0906** | **0.025** | **0.683** | **0.975** |
| **Smoking History:** |  |  | 0.85 |  |  |
| Never  (Baseline) |  |  |  |  |  |
| Former | 0.963 | 0.0675 | 0.581 | 0.844 | 1.1 |
| Current | 0.967 | 0.104 | 0.748 | 0.789 | 1.19 |
| **Physical Activity:** |  |  | **0.006** |  |  |
| Low  (Baseline) |  |  |  |  |  |
| Moderate | 0.972 | 0.0594 | 0.636 | 0.865 | 1.09 |
| Vigorous | **0.827** | **0.066** | **0.004** | **0.726** | **0.941** |
| Delayed Recall Score | **0.969** | **0.0154** | **0.043** | **0.941** | **0.999** |
| **Disabilities:** |  |  |  |  |  |
| 1+ ADLs | 1.15 | 0.111 | 0.204 | 0.927 | 1.43 |
| 1+ IADLS1 | 0.984 | 0.128 | 0.900 | 0.766 | 1.26 |
| **Time:** |  |  | **<0.001** |  |  |
| Wave 1  (Baseline) |  |  |  |  |  |
| Wave 3 | **1.13** | **0.0367** | **0.001** | **1.05** | **1.21** |
| Wave 5 | **1.22** | **0.0532** | **<0.001** | **1.1** | **1.35** |
| Number of comorbidities | **1.32** | **0.0244** | **< 0.001** | **1.26** | **1.39** |
| Sleep disturbance score | **1.04** | **0.0173** | **0.020** | **1.01** | **1.08** |
| **Chronic Pain** |  |  |  |  |  |
| No | 1.03 | 0.0585 | 0.612 | 0.919 | 1.16 |

*Supplementary Table S3: Results of survey weighted IPW-GEE to predict prevalence of arthritis*

|  | **Odds Ratio** | **Standard Error** | **p-value** | **95% CI Lower Limit** | **95% CI**  **Upper Limit** |
| --- | --- | --- | --- | --- | --- |
| (Intercept) | **0.00567** | **0.58** | **< 0.001** | **0.00182** | **0.0177** |
| Age | **1.05** | **0.00432** | **< 0.001** | **1.05** | **1.06** |
| BMI | **1.04** | **0.00715** | **< 0.001** | **1.03** | **1.06** |
| Waist Hip Ratio | 0.85 | 0.553 | 0.769 | 0.287 | 2.52 |
| **Sex:** |  |  |  |  |  |
| Female | **1.83** | **0.0884** | **< 0.001** | **1.54** | **2.17** |
| **Education:** |  |  | 0.22 |  |  |
| Primary or less  (Baseline) |  |  |  |  |  |
| Secondary | 0.922 | 0.0878 | 0.358 | 0.777 | 1.1 |
| Third Level | 1.05 | 0.0909 | 0.605 | 0.877 | 1.25 |
| **Smoking History:** |  |  | 0.076 |  |  |
| Never  (Baseline) |  |  |  |  |  |
| Former | **1.17** | **0.0699** | **0.025** | **1.02** | **1.34** |
| Current | 1.05 | 0.109 | 0.624 | 0.852 | 1.31 |
| **Physical Activity:** |  |  | 0.13 |  |  |
| Low  (Baseline) |  |  |  |  |  |
| Moderate | 0.899 | 0.0605 | 0.080 | 0.799 | 1.01 |
| Vigorous | 0.993 | 0.0683 | 0.923 | 0.869 | 1.14 |
| Delayed Recall Score | 0.977 | 0.0158 | 0.136 | 0.947 | 1.01 |
| **Disabilities:** |  |  |  |  |  |
| 1+ ADLs | **2.14** | **0.113** | **< 0.001** | **1.71** | **2.67** |
| 1+ IADLS1 | 1.04 | 0.132 | 0.752 | 0.804 | 1.35 |
| **Time:** |  |  | **<0.001** |  |  |
| Wave 1  (Baseline) |  |  |  |  |  |
| Wave 3 | **1.61** | **0.041** | **< 0.001** | **1.48** | **1.74** |
| Wave 5 | **1.67** | **0.0559** | **< 0.001** | **1.5** | **1.86** |
| Number of comorbidities | **1.08** | **0.0222** | **< 0.001** | **1.04** | **1.13** |
| Sleep disturbance score | **1.04** | **0.0181** | **0.018** | **1.01** | **1.08** |
| **Chronic Pain** |  |  |  |  |  |
| No | **0.267** | **0.0568** | **< 0.001** | **0.239** | **0.298** |

*Supplementary Table S4: Results of survey weighted IPW-GEE to predict prevalence of diabetes*

|  | **Estimate** | **Standard Error** | **p-value** | **95% CI Lower Limit** | **95% CI**  **Upper Limit** |
| --- | --- | --- | --- | --- | --- |
| (Intercept) | 0.00 | 0.98 | < 2e-16 | 0.00 | 0.00 |
| Age | **1.02** | **0.01** | **0.02** | **1.00** | **1.03** |
| BMI | **1.07** | **0.01** | **0.00** | **1.05** | **1.10** |
| Waist Hip Ratio | **1395** | **0.87** | **< 2e-16** | **255** | **7633** |
| **Sex:** |  |  |  |  |  |
| Female | 1.12 | 0.14 | 0.43 | 0.85 | 1.47 |
| **Education:** |  |  | 0.16 |  |  |
| Primary or less  (Baseline) |  |  |  |  |  |
| Secondary | 0.90 | 0.14 | 0.46 | 0.69 | 1.18 |
| Third Level | 1.15 | 0.14 | 0.33 | 0.87 | 1.52 |
| **Smoking History:** |  |  | **0.019** |  |  |
| Never  (Baseline) |  |  |  |  |  |
| Former | 0.96 | 0.12 | 0.74 | 0.77 | 1.21 |
| Current | **1.51** | **0.17** | **0.01** | **1.08** | **2.09** |
| **Physical Activity:** |  |  | **0.035** |  |  |
| Low  (Baseline) |  |  |  |  |  |
| Moderate | **0.88** | **0.10** | **0.20** | **0.73** | **1.07** |
| Vigorous | **0.75** | **0.11** | **0.01** | **0.61** | **0.93** |
| Delayed Recall Score |  |  |  |  |  |
| **Disabilities:** |  |  |  |  |  |
| 1+ ADLs | 1.10 | 0.15 | 0.54 | 0.81 | 1.48 |
| 1+ IADLS1 | 1.00 | 0.18 | 1.00 | 0.70 | 1.42 |
| **Time:** |  |  | **0.004** |  |  |
| Wave 1  (Baseline) |  |  |  |  |  |
| Wave 3 | **1.20** | **0.06** | **0.00** | **1.06** | **1.36** |
| Wave 5 | **1.32** | **0.09** | **0.00** | **1.11** | **1.57** |
| Number of comorbidities | **1.21** | **0.03** | **0.00** | **1.13** | **1.29** |
| Sleep disturbance score | **1.07** | **0.03** | **0.01** | **1.02** | **1.13** |
| **Chronic Pain** |  |  |  |  |  |
| No | 1.02 | 0.09 | 0.82 | 0.85 | 1.23 |

*Supplementary Table S5: Results of survey weighted IPW-GEE to predict prevalence of angina*

|  | **Odds Ratio** | **Standard Error** | **p-value** | **95% CI Lower Limit** | **95% CI**  **Upper Limit** |
| --- | --- | --- | --- | --- | --- |
| (Intercept) | **0.0001** | **1.23** | **1.30E-13** | **0.000009** | **0.0012** |
| Age | **1.08** | **0.00789** | **< 0.001** | **1.06** | **1.09** |
| BMI | **1.03** | **0.0117** | **0.011** | **1.01** | **1.05** |
| Waist Hip Ratio | 0.595 | 1.16 | 0.654 | 0.0615 | 5.76 |
| **Sex:** |  |  |  |  |  |
| Female | **0.349** | **0.192** | **< 0.001** | **0.24** | **0.509** |
| **Education:** |  |  | 0.055 |  |  |
| Primary or less  (Baseline) |  |  |  |  |  |
| Secondary | **1.64** | **0.151** | **0.001** | **1.22** | **2.21** |
| Third Level | 1.33 | 0.269 | 0.286 | 0.786 | 2.26 |
| **Smoking History:** |  |  | **0.004** |  |  |
| Never  (Baseline) |  |  |  |  |  |
| Former | 0.916 | 0.164 | 0.594 | 0.664 | 1.26 |
| Current | **0.658** | **0.183** | **0.022** | **0.46** | **0.941** |
| **Physical Activity:** |  |  | 0.8 |  |  |
| Low  (Baseline) |  |  |  |  |  |
| Moderate | 1.09 | 0.124 | 0.507 | 0.852 | 1.38 |
| Vigorous | 1.04 | 0.149 | 0.803 | 0.775 | 1.39 |
| Delayed Recall Score | 1.03 | 0.0318 | 0.325 | 0.969 | 1.1 |
| **Disabilities:** |  |  |  |  |  |
| 1+ ADLs | 1.17 | 0.183 | 0.4 | 0.815 | 1.67 |
| 1+ IADLS1 | 0.834 | 0.224 | 0.419 | 0.538 | 1.29 |
| **Time:** |  |  | **< 0.001** |  |  |
| Wave 1  (Baseline) |  |  |  |  |  |
| Wave 3 | **0.697** | **0.0884** | **< 0.001** | **0.586** | **0.829** |
| Wave 5 | **0.56** | **0.12** | **< 0.001** | **0.442** | **0.709** |
| Number of comorbidities | **1.49** | **0.0392** | **< 0.001** | **1.38** | **1.61** |
| Sleep disturbance score | **1.09** | **0.0352** | **0.017** | **1.02** | **1.17** |
| **Chronic Pain** |  |  |  |  |  |
| No | 0.811 | 0.117 | 0.074 | 0.644 | 1.02 |

*Supplementary Table S6: Results of survey weighted IPW-GEE to predict prevalence of respiratory disease*

|  | **Odds Ratio** | **Standard Error** | **p-value** | **95% CI Lower Limit** | **95% CI**  **Upper Limit** |
| --- | --- | --- | --- | --- | --- |
| (Intercept) | **0.0097** | **0.769** | **0.000** | **0.00215** | **0.0438** |
| Age | 0.994 | 0.00547 | 0.288 | 0.984 | 1 |
| BMI | 0.998 | 0.00947 | 0.850 | 0.98 | 1.02 |
| Waist Hip Ratio | **17.2** | **0.673** | **0.000** | **4.6** | **64.2** |
| **Sex:** |  |  |  |  |  |
| Female | **1.61** | **0.108** | **0.000** | **1.3** | **1.99** |
| **Education:** |  |  | **0.007** |  |  |
| Primary or less  (Baseline) |  |  |  |  |  |
| Secondary | **0.803** | **0.106** | **0.039** | **0.651** | **0.989** |
| Third Level | **0.705** | **0.112** | **0.002** | **0.566** | **0.877** |
| **Smoking History:** |  |  | **0.014** |  |  |
| Never  (Baseline) |  |  |  |  |  |
| Former | **1.3** | **0.0902** | **0.004** | **1.09** | **1.55** |
| Current | 1.2 | 0.128 | 0.162 | 0.93 | 1.54 |
| **Physical Activity:** |  |  | 0.82 |  |  |
| Low  (Baseline) |  |  |  |  |  |
| Moderate | 0.956 | 0.0763 | 0.558 | 0.824 | 1.11 |
| Vigorous | 0.957 | 0.0878 | 0.616 | 0.806 | 1.14 |
| Delayed Recall Score | 1.03 | 0.0199 | 0.185 | 0.987 | 1.07 |
| **Disabilities:** |  |  |  |  |  |
| 1+ ADLs | 1.25 | 0.124 | 0.076 | 0.978 | 1.59 |
| 1+ IADLS1 | **1.49** | **0.137** | **0.003** | **1.14** | **1.95** |
| **Time:** |  |  | **<0.001** |  |  |
| Wave 1  (Baseline) |  |  |  |  |  |
| Wave 3 | **1.18** | **0.0467** | **<0.001** | **1.08** | **1.29** |
| Wave 5 | **1.35** | **0.068** | **<0.001** | **1.18** | **1.54** |
| Number of comorbidities | **1.14** | **0.0267** | **<0.001** | **1.08** | **1.2** |
| Sleep disturbance score | **1.04** | **0.0218** | **0.049** | **1** | **1.09** |
| **Chronic Pain** |  |  |  |  |  |
| No | **0.8** | **0.0742** | **0.003** | **0.692** | **0.926** |

*Supplementary Table S7: Results of survey weighted IPW-GEE to predict prevalence of high cholesterol*

|  | **Odds Ratio** | **Standard Error** | **p-value** | **95% CI Lower Limit** | **95% CI**  **Upper Limit** |
| --- | --- | --- | --- | --- | --- |
| (Intercept) | **0.0376** | **0.543** | **0.000** | **0.013** | **0.109** |
| Age | **1.01** | **0.00419** | **0.001** | **1.01** | **1.02** |
| BMI | 1.01 | 0.00705 | 0.410 | 0.992 | 1.02 |
| Waist Hip Ratio | **5.54** | **0.507** | **0.001** | **2.05** | **14.9** |
| **Sex:** |  |  |  |  |  |
| Female | 1.09 | 0.0798 | 0.307 | 0.928 | 1.27 |
| **Education:** |  |  | **0.022** |  |  |
| Primary or less  (Baseline) |  |  |  |  |  |
| Secondary | 1.14 | 0.0834 | 0.108 | 0.971 | 1.35 |
| Third Level | **1.27** | **0.0863** | **0.006** | **1.07** | **1.5** |
| **Smoking History:** |  |  | 0.012 |  |  |
| Never  (Baseline) |  |  |  |  |  |
| Former | 1.1 | 0.0647 | 0.143 | 0.968 | 1.25 |
| Current | 0.931 | 0.0922 | 0.439 | 0.777 | 1.12 |
| **Physical Activity:** |  |  | **0.003** |  |  |
| Low  (Baseline) |  |  |  |  |  |
| Moderate | 0.974 | 0.0576 | 0.642 | 0.87 | 1.09 |
| Vigorous | **0.828** | **0.0621** | **0.002** | **0.733** | **0.935** |
| Delayed Recall Score | 1.01 | 0.0148 | 0.398 | 0.984 | 1.04 |
| **Disabilities:** |  |  |  |  |  |
| 1+ ADLs | 1.14 | 0.11 | 0.227 | 0.921 | 1.42 |
| 1+ IADLs | 0.914 | 0.128 | 0.479 | 0.711 | 1.17 |
| **Time:** |  |  | **<0.001** |  |  |
| Wave 1  (Baseline) |  |  |  |  |  |
| Wave 3 | **1.46** | **0.0352** | **<0.001** | **1.36** | **1.56** |
| Wave 5 | **1.61** | **0.0514** | **<0.001** | **1.46** | **1.78** |
| Number of comorbidities | **1.35** | **0.0251** | **<0.001** | **1.28** | **1.42** |
| Sleep disturbance score | 1.02 | 0.0166 | 0.199 | 0.989 | 1.06 |
| **Chronic Pain** |  |  |  |  |  |
| No | 0.912 | 0.0555 | 0.097 | 0.818 | 1.02 |

*Supplementary Table S8: Results of survey weighted IPW-GEE to predict prevalence of osteoporosis*

|  | **Estimate** | **Standard Error** | **p-value** | **95% CI Lower Limit** | **95% CI**  **Upper Limit** |
| --- | --- | --- | --- | --- | --- |
| (Intercept) | **0.0777** | **0.724** | **< 0.001** | **0.0188** | **0.321** |
| Age | **1.03** | **0.00523** | **< 0.001** | **1.02** | **1.04** |
| BMI | **0.903** | **0.0107** | **< 0.001** | **0.885** | **0.922** |
| Waist Hip Ratio | 1.21 | 0.672 | 0.772 | 0.325 | 4.53 |
| **Sex:** |  |  |  |  |  |
| Female | **7.14** | **0.124** | **< 0.001** | **5.6** | **9.11** |
| **Education:** |  |  |  |  |  |
| Primary or less  (Baseline) |  |  |  |  |  |
| Secondary | 1.06 | 0.113 | 0.584 | 0.853 | 1.33 |
| Third Level | **1.17** | **0.119** | 0.192 | **0.925** | **1.47** |
| **Smoking History:** |  |  |  |  |  |
| Never  (Baseline) |  |  |  |  |  |
| Former | 0.878 | 0.0858 | 0.129 | 0.742 | 1.04 |
| Current | **0.656** | **0.142** | **0.003** | **0.496** | **0.867** |
| **Physical Activity:** |  |  | 0.058 |  |  |
| Low  (Baseline) |  |  |  |  |  |
| Moderate | 0.865 | 0.075 | 0.053 | 0.747 | 1 |
| Vigorous | **0.825** | **0.0865** | **0.026** | **0.696** | **0.978** |
| Delayed Recall Score | **1** | **0.0198** | 0.976 | **0.963** | **1.04** |
| **Disabilities:** |  |  |  |  |  |
| 1+ ADLs | 0.955 | 0.136 | 0.735 | 0.732 | 1.25 |
| 1+ IADLS1 | **1.41** | **0.155** | **0.027** | **1.04** | **1.91** |
| **Time:** |  |  |  |  |  |
| Wave 1  (Baseline) |  |  |  |  |  |
| Wave 3 | **1.7** | **0.0505** | **< 0.001** | **1.54** | **1.87** |
| Wave 5 | **1.78** | **0.0663** | **< 0.001** | **1.56** | **2.03** |
| Number of comorbidities | **1.12** | **0.0275** | **< 0.001** | **1.06** | **1.18** |
| Sleep disturbance score | 1.02 | 0.0225 | 0.293 | 0.98 | 1.07 |
| **Chronic Pain** |  |  |  |  |  |
| No | **0.681** | **0.0724** | **< 0.001** | **0.591** | **0.785** |

*Supplementary Table S9: Results of survey weighted IPW-GEE to predict prevalence of heart attack*

|  | **Odds Ratio** | **Standard Error** | **p-value** | **95% CI Lower Limit** | **95% CI**  **Upper Limit** |
| --- | --- | --- | --- | --- | --- |
| (Intercept) | **0.00033** | **1.39** | **<0.001** | **2.17E-05** | **0.00502** |
| Age | **1.04** | **0.00968** | **<0.001** | **1.02** | **1.06** |
| BMI | **1.03** | **0.013** | **0.017** | **1.01** | **1.06** |
| Waist Hip Ratio | 2.51 | 1.21 | 0.445 | 0.237 | 26.7 |
| **Sex:** |  |  |  |  |  |
| Female | **0.214** | **0.214** | **<0.001** | **0.141** | **0.326** |
| **Education:** |  |  | 0.87 |  |  |
| Primary or less  (Baseline) |  |  |  |  |  |
| Secondary | 0.959 | 0.179 | 0.815 | 0.675 | 1.36 |
| Third Level | 0.906 | 0.191 | 0.605 | 0.623 | 1.32 |
| **Smoking History:** |  |  | **<0.001** |  |  |
| Never  (Baseline) |  |  |  |  |  |
| Former | **2.12** | **0.169** | **<0.001** | **1.52** | **2.96** |
| Current | **1.99** | **0.237** | **0.004** | **1.25** | **3.16** |
| **Physical Activity:** |  |  | 0.37 |  |  |
| Low  (Baseline) |  |  |  |  |  |
| Moderate | 0.918 | 0.131 | 0.515 | 0.71 | 1.19 |
| Vigorous | 0.794 | 0.164 | 0.159 | 0.576 | 1.09 |
| Delayed Recall Score | 0.949 | 0.0395 | 0.184 | 0.878 | 1.03 |
| **Disabilities:** |  |  |  |  |  |
| 1+ ADLs | 0.937 | 0.203 | 0.749 | 0.63 | 1.39 |
| 1+ IADLS1 | 0.859 | 0.258 | 0.556 | 0.518 | 1.42 |
| **Time:** |  |  | **<0.001** |  |  |
| Wave 1  (Baseline) |  |  |  |  |  |
| Wave 3 | **0.728** | **0.0892** | **<0.001** | **0.612** | **0.868** |
| Wave 5 | **0.573** | **0.132** | **<0.001** | **0.443** | **0.741** |
| Number of comorbidities | **1.43** | **0.0434** | **<0.001** | **1.31** | **1.55** |
| Sleep disturbance score | 0.988 | 0.0405 | 0.759 | 0.912 | 1.07 |
| **Chronic Pain** |  |  |  |  |  |
| No | 1.22 | 0.124 | 0.107 | 0.958 | 1.56 |

**Appendix 6: Causal Mediation Analysis Results**

*Supplementary Table S10: High Cholesterol Causal Mediation Results*

|  | **Estimate 95%** | **CI Lower 95%** | **CI Upper 95%** | **p-value** |
| --- | --- | --- | --- | --- |
| *Low Vs Vigorous Physical Activity* |  |  |  |  |
| ACME (control) | -0.001 | -0.002 | 0.00 | <0.001 |
| ACME (treated) | -0.001 | -0.002 | 0.00 | <0.001 |
| ADE (control) | -0.038 | -0.058 | -0.02 | <0.001 |
| ADE (treated) | -0.038 | -0.058 | -0.02 | <0.001 |
| Total Effect | -0.039 | -0.060 | -0.02 | <0.001 |
| Prop. Mediated (control) | 0.032 | 0.009 | 0.09 | <0.001 |
| Prop. Mediated (treated) | 0.033 | 0.009 | 0.09 | <0.001 |
| ACME (average) | -0.001 | -0.002 | 0.00 | <0.001 |
| ADE (average) | -0.038 | -0.058 | -0.02 | <0.001 |
| Prop. Mediated (average) | 0.032 | 0.009 | 0.09 | <0.001 |
| *Moderate Vs Vigorous Physical Activity* |  |  |  |  |
| ACME (control) | -0.001 | -0.002 | 0 | 0.002 |
| ACME (treated) | -0.001 | -0.002 | 0 | 0.002 |
| ADE (control) | -0.028 | -0.048 | -0.01 | <0.001 |
| ADE (treated) | -0.028 | -0.048 | -0.01 | <0.001 |
| Total Effect | -0.029 | -0.049 | -0.01 | <0.001 |
| Prop. Mediated (control) | 0.033 | 0.009 | 0.12 | 0.002 |
| Prop. Mediated (treated) | 0.034 | 0.009 | 0.12 | 0.002 |
| ACME (average) | -0.001 | -0.002 | 0 | 0.002 |
| ADE (average) | -0.028 | -0.048 | -0.01 | <0.001 |
| Prop. Mediated (average) | 0.033 | 0.009 | 0.12 | 0.002 |
| *Low Vs Moderate* |  |  |  |  |
| ACME (control) | 0.000 | -0.001 | 0 | 0.33 |
| ACME (treated) | 0.000 | -0.001 | 0 | 0.33 |
| ADE (control) | -0.009 | -0.028 | 0.01 | 0.34 |
| ADE (treated) | -0.009 | -0.028 | 0.01 | 0.34 |
| Total Effect | -0.010 | -0.028 | 0.01 | 0.34 |
| Prop. Mediated (control) | 0.014 | -0.271 | 0.22 | 0.58 |
| Prop. Mediated (treated) | 0.014 | -0.270 | 0.22 | 0.58 |
| ACME (average) | 0.000 | -0.001 | 0 | 0.33 |
| ADE (average) | -0.009 | -0.028 | 0.01 | 0.34 |
| Prop. Mediated (average) | 0.014 | -0.270 | 0.22 | 0.58 |

*Supplementary Table S11: Hypertension Causal Mediation Results*

|  | **Estimate 95%** | **CI Lower 95%** | **CI Upper 95%** | **p-value** |
| --- | --- | --- | --- | --- |
| *Low Vs Vigorous Physical Activity* |  |  |  |  |
| ACME (control) | -0.001 | -0.002 | 0.000 | 0.010 |
| ACME (treated) | -0.001 | -0.002 | 0.000 | 0.010 |
| ADE (control) | -0.038 | -0.058 | -0.020 | 0.002 |
| ADE (treated) | -0.038 | -0.058 | -0.020 | 0.002 |
| Total Effect | -0.039 | -0.059 | -0.020 | 0.002 |
| Prop. Mediated (control) | 0.027 | 0.006 | 0.070 | 0.012 |
| Prop. Mediated (treated) | 0.027 | 0.006 | 0.070 | 0.012 |
| ACME (average) | -0.001 | -0.002 | 0.000 | 0.010 |
| ADE (average) | -0.038 | -0.058 | -0.020 | 0.002 |
| Prop. Mediated (average) | 0.027 | 0.006 | 0.070 | 0.012 |
| *Moderate Vs Vigorous Physical Activity* |  |  |  |  |
| ACME (control) | -0.001 | -0.002 | 0.000 | 0.006 |
| ACME (treated) | -0.001 | -0.002 | 0.000 | 0.006 |
| ADE (control) | -0.032 | -0.049 | -0.010 | 0.002 |
| ADE (treated) | -0.032 | -0.049 | -0.010 | 0.002 |
| Total Effect | -0.033 | -0.051 | -0.010 | 0.002 |
| Prop. Mediated (control) | 0.025 | 0.006 | 0.070 | 0.008 |
| Prop. Mediated (treated) | 0.025 | 0.006 | 0.070 | 0.008 |
| ACME (average) | -0.001 | -0.002 | 0.000 | 0.006 |
| ADE (average) | -0.032 | -0.049 | -0.010 | 0.002 |
| Prop. Mediated (average) | 0.025 | 0.006 | 0.070 | 0.008 |
| *Low Vs Moderate* |  |  |  |  |
| ACME (control) | 0.000 | -0.001 | 0.000 | 0.330 |
| ACME (treated) | 0.000 | -0.001 | 0.000 | 0.330 |
| ADE (control) | -0.006 | -0.025 | 0.010 | 0.550 |
| ADE (treated) | -0.006 | -0.025 | 0.010 | 0.550 |
| Total Effect | -0.006 | -0.025 | 0.010 | 0.540 |
| Prop. Mediated (control) | 0.010 | -0.433 | 0.260 | 0.690 |
| Prop. Mediated (treated) | 0.010 | -0.433 | 0.260 | 0.690 |
| ACME (average) | 0.000 | -0.001 | 0.000 | 0.330 |
| ADE (average) | -0.006 | -0.025 | 0.010 | 0.550 |
| Prop. Mediated (average) | 0.010 | -0.433 | 0.260 | 0.690 |

*Supplementary Table S12: Diabetes Causal Mediation Results*

|  | **Estimate 95%** | **CI Lower 95%** | **CI Upper 95%** | **p-value** |
| --- | --- | --- | --- | --- |
| *Low Vs Vigorous Physical Activity* |  |  |  |  |
| ACME (control) | -0.001 | -0.002 | 0.000 | 0.012 |
| ACME (treated) | -0.001 | -0.002 | 0.000 | 0.012 |
| ADE (control) | -0.021 | -0.036 | -0.010 | 0.012 |
| ADE (treated) | -0.021 | -0.036 | -0.010 | 0.012 |
| Total Effect | -0.022 | -0.037 | -0.010 | 0.012 |
| Prop. Mediated (control) | 0.041 | 0.006 | 0.130 | 0.024 |
| Prop. Mediated (treated) | 0.033 | 0.005 | 0.120 | 0.024 |
| ACME (average) | -0.001 | -0.002 | 0.000 | 0.012 |
| ADE (average) | -0.021 | -0.036 | -0.010 | 0.012 |
| Prop. Mediated (average) | 0.037 | 0.005 | 0.120 | 0.024 |
| *Moderate Vs Vigorous Physical Activity* |  |  |  |  |
| ACME (control) | -0.001 | -0.002 | 0.000 | 0.008 |
| ACME (treated) | -0.001 | -0.002 | 0.000 | 0.008 |
| ADE (control) | -0.011 | -0.027 | 0.000 | 0.166 |
| ADE (treated) | -0.011 | -0.027 | 0.000 | 0.166 |
| Total Effect | -0.012 | -0.028 | 0.000 | 0.140 |
| Prop. Mediated (control) | 0.059 | -0.516 | 0.630 | 0.144 |
| Prop. Mediated (treated) | 0.052 | -0.527 | 0.620 | 0.144 |
| ACME (average) | -0.001 | -0.002 | 0.000 | 0.008 |
| ADE (average) | -0.011 | -0.027 | 0.000 | 0.166 |
| Prop. Mediated (average) | 0.056 | -0.521 | 0.630 | 0.144 |
| *Low Vs Moderate* |  |  |  |  |
| ACME (control) | 0.000 | -0.001 | 0.000 | 0.330 |
| ACME (treated) | 0.000 | -0.001 | 0.000 | 0.330 |
| ADE (control) | -0.009 | -0.020 | 0.000 | 0.120 |
| ADE (treated) | -0.009 | -0.020 | 0.000 | 0.120 |
| Total Effect | -0.009 | -0.020 | 0.000 | 0.110 |
| Prop. Mediated (control) | 0.013 | -0.091 | 0.140 | 0.420 |
| Prop. Mediated (treated) | 0.012 | -0.093 | 0.140 | 0.420 |
| ACME (average) | 0.000 | -0.001 | 0.000 | 0.330 |
| ADE (average) | -0.009 | -0.020 | 0.000 | 0.120 |
| Prop. Mediated (average) | 0.012 | -0.092 | 0.140 | 0.420 |

*Supplementary Table S13: Osteoporosis Causal Mediation Results*

|  | **Estimate 95%** | **CI Lower 95%** | **CI Upper 95%** | **p-value** |
| --- | --- | --- | --- | --- |
| *Low Vs Vigorous Physical Activity* |  |  |  |  |
| ACME (control) | -0.001 | -0.002 | 0.000 | <0.001 |
| ACME (treated) | -0.001 | -0.002 | 0.000 | <0.001 |
| ADE (control) | -0.021 | -0.037 | -0.010 | 0.012 |
| ADE (treated) | -0.021 | -0.037 | -0.010 | 0.012 |
| Total Effect | -0.022 | -0.038 | -0.010 | 0.006 |
| Prop. Mediated (control) | 0.044 | 0.015 | 0.160 | 0.006 |
| Prop. Mediated (treated) | 0.041 | 0.013 | 0.150 | 0.006 |
| ACME (average) | -0.001 | -0.002 | 0.000 | <0.001 |
| ADE (average) | -0.021 | -0.037 | -0.010 | 0.012 |
| Prop. Mediated (average) | 0.043 | 0.014 | 0.160 | 0.006 |
| *Moderate Vs Vigorous Physical Activity* |  |  |  |  |
| ACME (control) | -0.001 | -0.002 | 0.000 | <0.001 |
| ACME (treated) | -0.001 | -0.001 | 0.000 | <0.001 |
| ADE (control) | -0.007 | -0.022 | 0.010 | 0.350 |
| ADE (treated) | -0.007 | -0.022 | 0.010 | 0.350 |
| Total Effect | -0.008 | -0.023 | 0.010 | 0.300 |
| Prop. Mediated (control) | 0.064 | -0.649 | 0.900 | 0.300 |
| Prop. Mediated (treated) | 0.061 | -0.654 | 0.900 | 0.300 |
| ACME (average) | -0.001 | -0.002 | 0.000 | <0.001 |
| ADE (average) | -0.007 | -0.022 | 0.010 | 0.350 |
| Prop. Mediated (average) | 0.062 | -0.652 | 0.900 | 0.300 |
| *Low Vs Moderate* |  |  |  |  |
| ACME (control) | 0.000 | -0.001 | 0.000 | 0.278 |
| ACME (treated) | 0.000 | -0.001 | 0.000 | 0.278 |
| ADE (control) | -0.014 | -0.028 | 0.000 | 0.056 |
| ADE (treated) | -0.014 | -0.028 | 0.000 | 0.056 |
| Total Effect | -0.014 | -0.028 | 0.000 | 0.048 |
| Prop. Mediated (control) | 0.015 | -0.040 | 0.130 | 0.314 |
| Prop. Mediated (treated) | 0.014 | -0.039 | 0.130 | 0.314 |
| ACME (average) | 0.000 | -0.001 | 0.000 | 0.278 |
| ADE (average) | -0.014 | -0.028 | 0.000 | 0.056 |
| Prop. Mediated (average) | 0.015 | -0.039 | 0.130 | 0.314 |
